# Disentangling Species Delineation and Guiding Conservation of Endangered Magnolias in Veracruz, Mexico

**DOI:** 10.3390/plants10040673

**Published:** 2021-03-31

**Authors:** Fabián Augusto Aldaba Núñez, Emily Veltjen, Esteban Manuel Martínez Salas, Marie-Stéphanie Samain

**Affiliations:** 1 Instituto de Ecología, A.C., Red de Diversidad Biológica del Occidente Mexicano, Pátzcuaro 61600, Mexico; mariestephanie.samain@gmail.com; 2 Systematic and Evolutionary Botany Lab, Department of Biology, Ghent University, 9000 Ghent, Belgium; emily.veltjen@ugent.be; 3 Ghent University Botanical Garden, Ghent University, 9000 Ghent, Belgium; 4 Herbario Nacional de México, Departamento de Botánica, Instituto de Biología, Universidad Nacional Autónoma de México, Mexico City 04510, Mexico; ems@ib.unam.mx

**Keywords:** conservation units, genetic diversity, IUCN Red List conservation status, Magnoliaceae, microsatellite, neotropical trees, SSR, *Talauma*

## Abstract

The Mexican state of Veracruz has suffered very high deforestation rates in the last few decades, and despite the establishment of protected areas and conservation projects, primary forest is now mainly persisting in mostly small, scattered, fragmented remnants. New species of *Magnolia* section *Talauma* in this state have been described with little to no reference to the already existing ones, potentially resulting in over-splitting, obscuring their taxonomic delineation and conservation status, and consequently conservation programs. To study the conservation units and their genetic diversity, we here employ 15 microsatellite markers on a highly representative sampling of 254 individuals of what are presumed to be five *Magnolia* species. The results support at least three species and maximum five main conservation units. We propose downgrading the latter to four, given morphological, ecological, demographical, and geographical considerations. Two out of the three sympatrically occurring species in the rainforest in the Los Tuxtlas volcanic area have weak genetic evidence to be considered separate species. Similarly, the individuals in the Sierra de Zongolica in central Veracruz, who bear a very high morphological and genetic similarity to *Magnolia mexicana*, have weak genetic evidence to be recognised as a separate species. Nonetheless, the individuals could be identified as *Magnolia decastroi* based on morphology, and further research including the full range of this species is recommended.

## 1. Introduction

Biodiversity is being lost at an accelerated rate, often referred to as the sixth mass extinction [1,2]. This is particularly striking in plant diverse countries such as Mexico, which span a wide variety of ecosystems [3,4]. The latest assessment of plant richness in Mexico registers 297 families, 2854 genera, and 23,314 species, of which 11,600 are endemic [5]. Particularly in the state of Veracruz, there are 271 families, 1956 genera, and 8497 vascular plant species, of which 238 are endemics, representing around 27% of the national diversity, being the third most diverse state in terms of plants [5,6,7]. This plant biodiversity is threatened mainly by land conversion, which has resulted in the loss of 42% of the tropical ecosystems [3]. The state of Veracruz ranks first nationally in the loss of vegetation; besides, it is estimated that only 8.6% of this vegetation is conserved [8], which is mainly found in unprotected areas [7]. Within Veracruz, the areas of Sierra de Zongolica and Los Tuxtlas have been recognised for their great biological and ecological diversity [7,9,10,11,12], which in recent decades have been largely destroyed, mainly by primary economic activities (agriculture and livestock), despite the fact that a large part of the territory of Los Tuxtlas is formally protected [9,11,13,14]. Therefore, any study carried out in these areas is of vital importance in order to propose conservation strategies that mitigate the effects of anthropogenic development.

Representatives of the Magnoliaceae family are part of these threatened, small, declining tropical ecosystems in Veracruz, and have great potential to serve as flagship [15] and umbrella [16,17] species for conservation studies and management. The first is due to their striking flowers and interesting evolutionary history [18,19,20]. Their status as umbrella species owes to the fact that these trees are one of the main constituents of their forest ecosystem. Since [16] highlighted the urgent need for conservation work based on genetic research in Magnoliaceae, focus on Neotropical Magnolias is increasing, with very promising and hopeful results for the species and forest conservation [21,22,23]. Although certain progress is being made, less than 50% of the most threatened taxa are found in ex situ collections in botanical gardens and arboreta [24], and more research is needed on (Critically) Endangered species. To allow for effective conservation, there are three basic steps [16]: (1) delimitation and selection of priority taxa; (2) analysis of diversity of natural populations and ex situ collections, and (3) final selection of sampling sites.

Delimitation of taxa, the initial action of the abovementioned steps, is especially challenging for the Magnolias of Veracruz belonging to the genus *Magnolia* sect. *Talauma sensu* Figlar and Nooteboom [25]. This concerns the three recently described endemic and endangered species that occur in the region of Los Tuxtlas: *Magnolia lopezobradorii* A. Vázquez, *Magnolia sinacacolinii* A. Vázquez and *Magnolia zoquepopolucae* A. Vázquez; one in the Sierra de Zongolica area in Southern Veracruz: *Magnolia decastroi* A. Vázquez and M. Muñiz and one in the Uxpanapa area in Southern Veracruz: *Magnolia wendtii* A. Vázquez [26,27,28]. The first three species had been assessed as Data Deficient (DD) by the International Union for Conservation of Nature (IUCN) Red List, because they lacked population information and there were doubts about their taxonomic status. In contrast, *M. decastroi* was assessed as Endangered (EN) and *M. wendtii* as Critically Endangered (CR). These five species were segregated from *Magnolia mexicana* DC., previously considered to be widely distributed in Mexico: from Veracruz to Chiapas in the east and from Jalisco to Guerrero in Western Mexico [29]. There are even specimens from Guatemala identified as *M. mexicana* [30]. *Magnolia mexicana* s.s. (not including the five segregated species) was assessed as Vulnerable [31]. Most of these Magnolias have local uses that are threatening their survival, either through logging for construction of fence doors (*M. sinacacolinii*), construction of houses and furniture (*M. zoquepopolucae*), and collection of complete flowers for medicinal application against heart diseases (*M. mexicana*) (pers. obs.).

As a result of the very short protologue descriptions of four of the five recently described section *Talauma* species in Veracruz and the often contradicting carpel numbers in the available identification keys [32,33], their circumscription is no longer clear. Moreover, some of the species descriptions are based on few specimens [27,28], or in the case of *M. mexicana* that was described in 1893, it is based on a scientific illustration [34,35,36,37]. The concept of *M. mexicana* in particular becomes even more challenging, because the scientific illustration is said to be originating from Cuernavaca, in the state of Morelos in Central-Southern Mexico [34,37], yet it is known that *M. mexicana* is not native to this area. Hence, it is most likely a specimen cultivated by the Aztec culture due to its medicinal properties. Furthermore, the illustration nor the description mention some of the characteristics of the section *Talauma*, such as the stipule scar along the entire length of the petiole or the circumcised fruit dehiscence [7,29,38], together with leaf and petal morphological characteristics that have been observed in living specimens.

When the identity and relationships are complex, morphological data can be complemented with molecular characters [39,40]. Including both types of data offers a closer approximation to the relationships between them, whereby conflicts between the two can be resolved by analysis of total evidence [41]. Recently, SSR (Single Sequence Repeat) markers have been used to elucidate the genetic patterns of Neotropical Magnolias in fragmented environments and the data obtained have shown that these species still have ample gene flow within populations, yet little gene flow between populations [42].

These five species have not been studied from a molecular point of view (e.g., phylogenetically): only *M. decastroi* and *M. mexicana* have been poorly studied in Mexico, sometimes with a minimum sample size [43,44] and *M. mexicana* from one accession is sequenced in family-wide studies [18,19,20,45,46], but those identifications have a high probability of being incorrect given the former widespread concept of the species. In a first taxonomic review [47], morphological characteristics of around 300 accessions of these species were tested statistically for their morphological distinctness, and here no significant morphological differences were found between *M. lopezobradorii* and *M. zoquepopolucae*.

**Figure 1:**
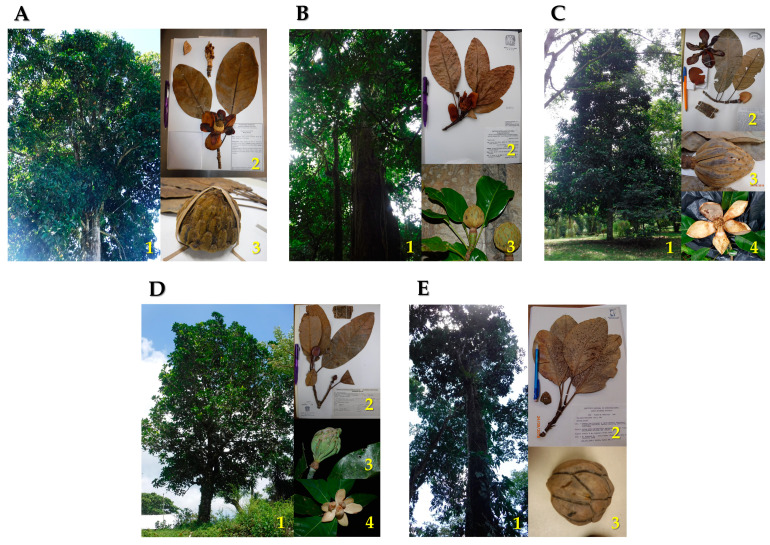
Morphology of *Magnolia* sect. *Talauma* in Puebla and Veracruz, Mexico. (**A**) *Magnolia decastroi*: 1. Tree, 2. Voucher, 3. Fruit; 1–3 By F.A. Aldaba Núñez, 2019. (**B**) *Magnolia lopezobradorii*: 1. Tree, 2. Voucher, 3. Fruit; 1–2 By F.A. Aldaba Núñez, 2019, 3 By E.M. Martínez Salas, 2019. (**C**) *Magnolia mexicana*: 1. Tree, 2. Voucher, 3. Fruit, 4. Flower; 1–4 By F.A. Aldaba Núñez, 2019. (**D**) *Magnolia sinacacolinii*: 1. Tree, 2. Voucher, 3. Fruit, 4. Flower; 1–2 By F.A. Aldaba Núñez, 2019, 3–4 By E.M. Martínez Salas, 2019. (**E**) *Magnolia zoquepopolucae*: 1. Tree, 2. Voucher, 3. Fruit; 1–3 By F.A. Aldaba Núñez, 2019.

**Figure 2:**
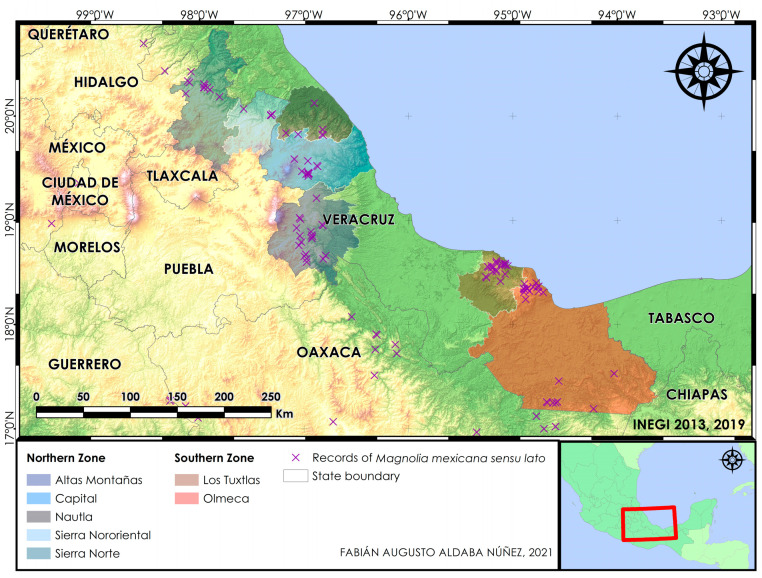
Map of study zones showing their natural regions; in Puebla and Veracruz states.

Here we aimed to assess the genetic diversity and structure of five *Magnolia* species (Figure 1) from Veracruz and surrounding regions, especially the state of Puebla (Figure 2), namely, *M. decastroi*, *M. lopezobradorii*, *M. mexicana*, *M. sinacacolinii*, and *M. zoquepopolucae*, employing 15 SSR markers, with the applied goal of developing effective conservation strategies tailored for the sampled localities under study. We specifically wanted to test: (1) do the SSR data support the five morphospecies?, (2) are the individuals at the different localities within the taxa exhibiting sufficient (past) gene flow and random mating to maintain (relatively to other studied Magnolias) healthy levels of genetic diversity?, (3) what should we consider the conservation units?, (4) which are priority taxa/localities for conservation management?, and (5) are the current ex situ collections a good genetic representation of the in situ diversity? The acquired insights of the demographic, distributional, genetic, morphological, and habitat data allowed us to re-assess their conservation status for the IUCN Red List, as well as to propose conservation strategies for each of the species.

## 2. Results

### 2.1. Genetic Analysis and Characterisation

#### 2.1.1. Null Alleles and Linkage Disequilibrium

In the analyses respecting the localities, several potential null-alleles were reported for five of the 15 SSR markers (Table 1). The locality FA-6 had potential null-alleles in four markers, and hence we suspect that the markers MA39_009, MA39_287, and MA42_421, which only had a potential null-allele detected for that locality, do not have true null-alleles. Instead, the strong signal of deviations of Hardy–Weinberg Proportions (HWP) used to determine null-allele presence comes forth from an underlying biological reason or sampling issues linked to this locality [48]. We do acknowledge that there is a chance that the null alleles are species-specific for FA-6, which is identified as *M. sinacacolinii*, as we only have one locality with a sample size N > 10 to study the presence of null alleles for that species. For locus MA42_471, two out of the 11 localities showed that the marker might express null alleles with null allele frequencies being 0.061 and 0.011 for the localities FA-12 (*M. decastroi*) and FA-10 (*M. mexicana*), respectively. However, because the two species have parallel, larger (N > 10) sampled localities, in which null alleles are not detected, and as the null allele frequencies are low, we decided to keep the markers in the subsequent analyses. Marker MA39_224 yielded null alleles for all study localities, with estimated frequencies between 0.122 and 0.390. As this pattern is consistent and the estimated null allele frequencies are high, we deleted marker MA39_224 from subsequent analyses and continued the downstream analyses with 14 SSR markers.

In the analyses for Linkage Disequilibrium (LD), we found 161 out of 1 108 significant pairwise comparisons (all pairwise tests that could not be performed due to low allelic variation or small sample sizes were not included to determine the total number of pairwise comparisons). For 1 108 pairwise tests we expected there to be 55.4 [44,49] Type I errors on the nominal p-level of 5%, hence linkage was at hand in the dataset. Following sequential Bonferroni correction, six of the pairwise comparisons remained in LD. Five of the six pairs were between MA40_282 and MA39_236; and one was between MA40_282 and MA42_495. We removed marker MA40_282 from the dataset as it cannot be guaranteed that this marker is an independent sampling of the genome with respect to the other SSR markers. We thus executed further downstream analyses with 13 microsatellite markers.

#### 2.1.2. Genetic Structure

**Figure 3:**
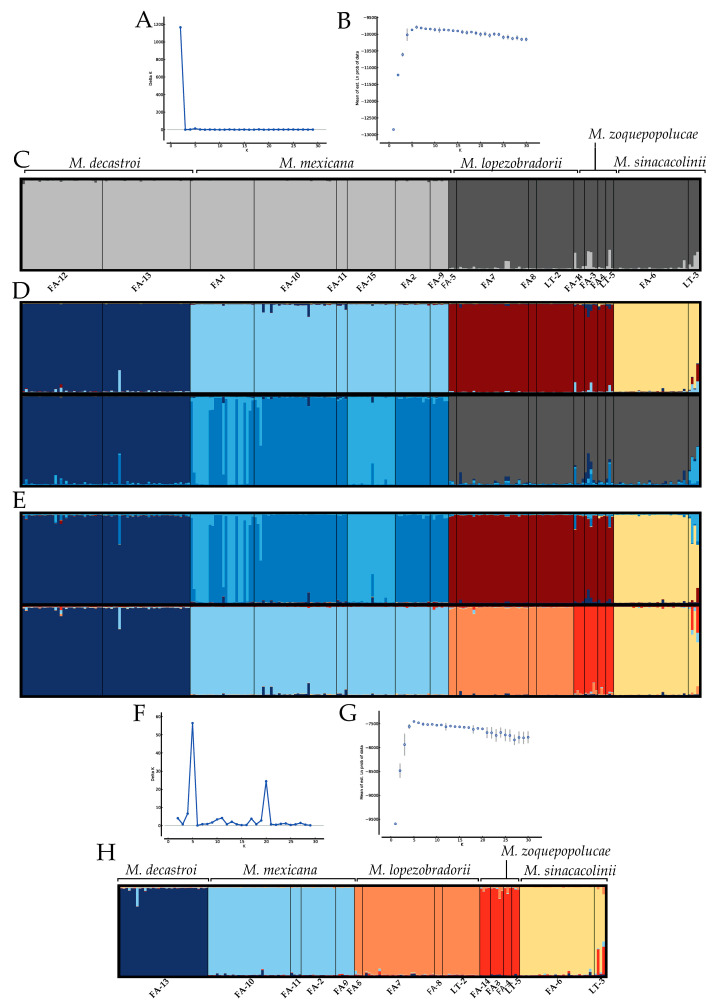
Structure bar plots of *Magnolia* sect. *Talauma* individuals in Puebla and Veracruz, Mexico. A–E: analyses on the complete dataset of 18 localities. F–H: analyses of the 15 wild localities only. (**A**) The delta K plot for the complete dataset. (**B**) The mean Ln(K) plot for the complete dataset. (**C**) Representative bar plot (out of ten replicates) for K = 2. (**D**) Representative bar plots for K = 4. The upper bar plot is found in 9/10 replicates, the lower bar plot in 1/10 replicates. (**E**) Representative bar plots for K = 5. The upper bar plot is found in 8/10 replicates, the lower bar plot in 2/10 replicates. (**F**) The delta K plot for the wild localities only. (**G**) The mean Ln(K) plot for the wild localities only. (**H**) Representative bar plot (out of ten replicates) for K = 5.

**Figure A1:**
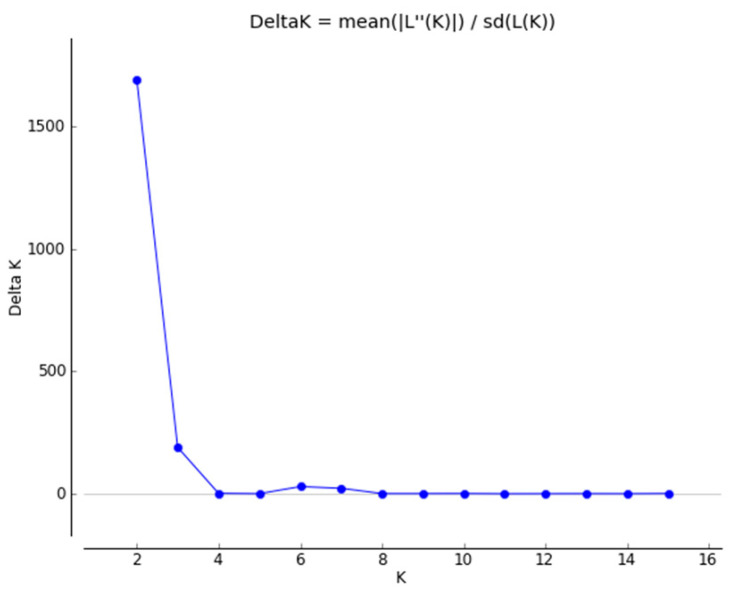
Delta K plot for the Northern Zone.

**Figure A2:**
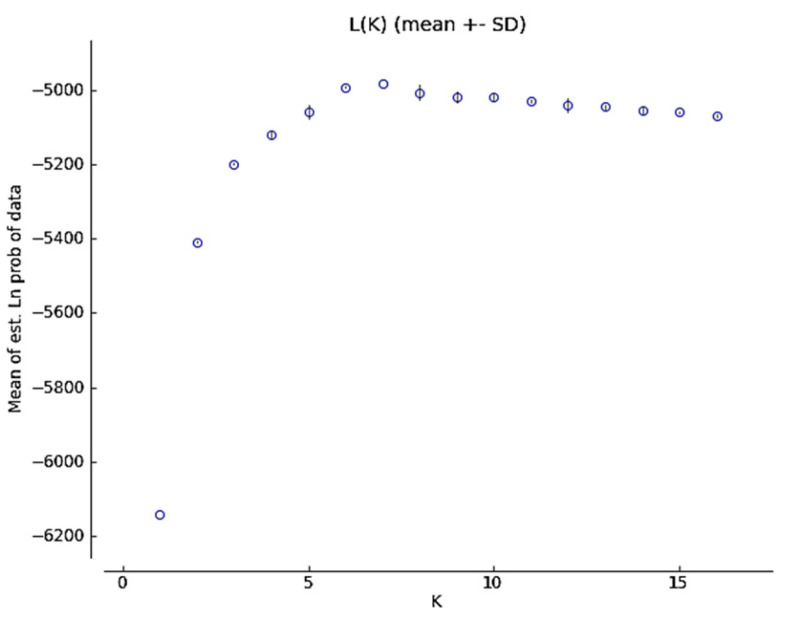
Mean Ln(K) plot for the Northern Zone.

**Figure A3:**
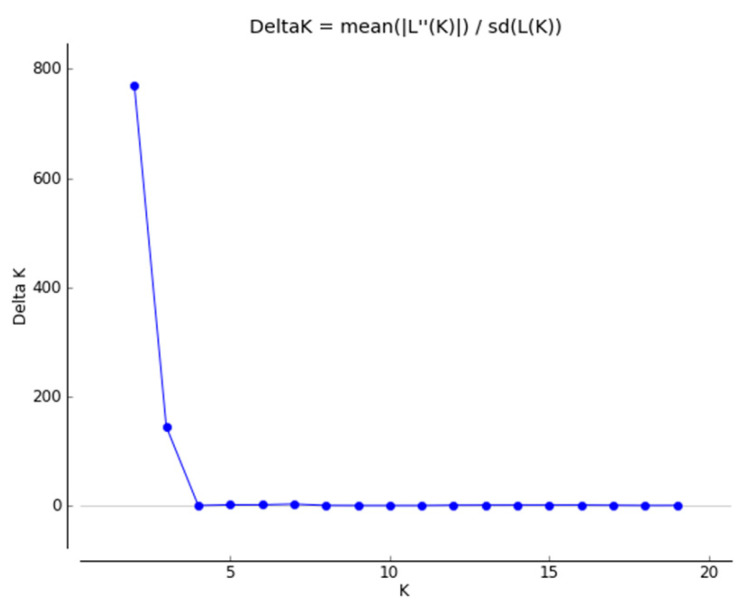
Delta K plot for the Southern Zone.

**Figure A4:**
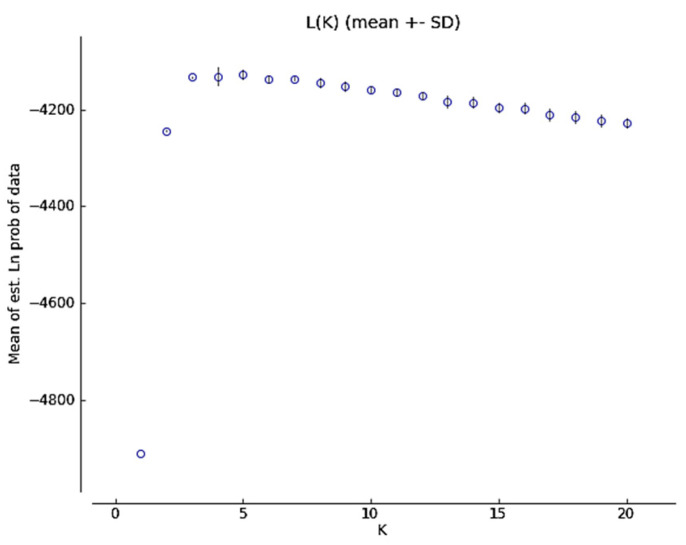
Mean Ln(K) plot for the Southern Zone.

According to the Structure analyses, the optimal ΔK value for the complete dataset was K = 2 (Figure 3A), which separates the 18 localities according to the two main sampled zones: the Northern Zone and the Southern Zone (Figure 2). The mean L(K) graph (Figure 3B) illustrates that the likelihood increases substantially when further subdivision is allowed at K = 3–5, and we expected 4-5 species based on [47]; hence, we explored the bar plots K = 3–5 in greater detail. The bar plot for K = 2 of the complete dataset is visualised in Figure 3C. When we studied the ten replicate bar plots from K=3, the 10 replicates were the following: 4 replicates clustered localities of *M. decastroi*, *M. mexicana* and the Southern Zone; 4 replicates clustered the localities of the Northern Zone, *M. lopezobradorii*–*M. zoquepopolucae* and *M. sinacacolinii*; 2 replicates clustered *M. decastroi*–*M. sinacacolinii*, *M. mexicana* and *M. lopezobradorii*–*M. zoquepopolucae*. When we studied the ten replicates from K = 4 and K = 5 (visualized in Figure 3D,E), 9/10 and 2/10 replicates indicate clusters according to species boundaries, respectively with a "conflicting" signal of individuals in the *M. mexicana* localities. When analyzing the Northern Zone and the Southern Zone separately in two new Structure analyses, the results are as follows. The optimal ΔK value for the Northern Zone was K = 2 (Figure A1), supported by the Mean L(K) plot result (Figure A2), splitting the *M. decastroi* and *M. mexicana* localities. The optimal ΔK value for the Southern Zone was K = 2 (Figure A3), supported by the Mean L(K) plot result (Figure A4), separating the *M. lopezobradorii* and *M. zoquepopolucae* localities from the *M. sinacacolinii* localities. For the dataset only comprising the wild localities, the optimal ΔK value was K=5 (Figure 3F), separating the 15 localities according to the previously defined species (Figure 3H).

**Figure 4:**
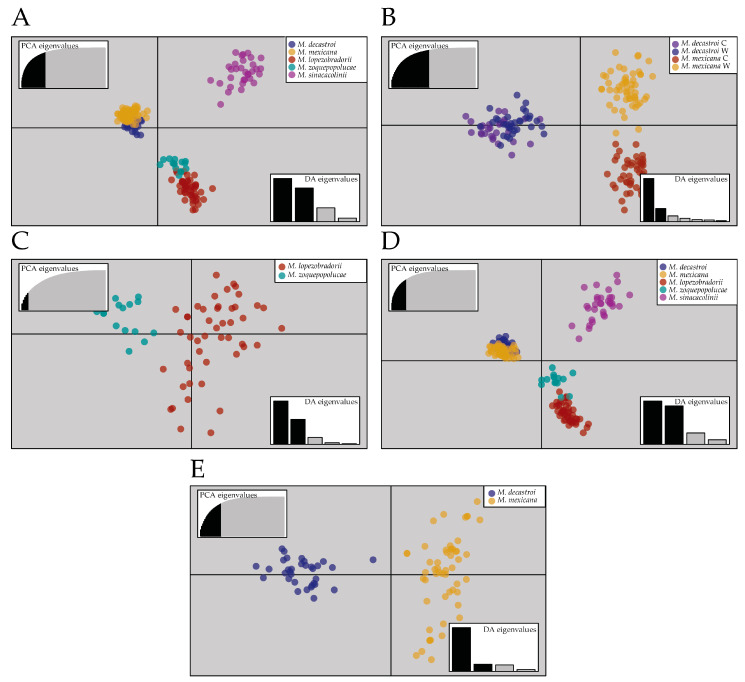
Discriminant Analysis of Principal Components (DAPC) of *Magnolia* sect. *Talauma* individuals in Puebla and Veracruz, Mexico. The axes represent the first two linear discriminants. The upper left graph (principal component analysis (PCA) eigenvalues) inset displays the variance explained by the principal component axes used for DAPC and the bottom-right inset (DA eigenvalues) displays in relative magnitude the variance explained by the two discriminant axes plotted. (**A**) DAPC graph of the complete dataset analysis, 150 principal components (PCs) retained. (**B**) DAPC graph of the *M. decastroi* and *M. mexicana* localities, 50 PCs retained. (**C**)**.** DAPC graph of the *M. lopezobradorii* and *M. zoquepopolucae* localities, 5 PCs retained. (**D**) DAPC graph of the wild dataset, 150 PCs retained. (**E**) DAPC graph of the *M. decastroi* and *M. mexicana* wild localities, 60 PCs retained.

Results from the DAPC analysis from the complete dataset (Figure 3A) revealed three main groups: the localities containing individuals identified as *M. decastroi*–*M. mexicana* and *M. lopezobradorii*–*M. zoquepopolucae* were clustered closely together according to the two most explanatory axes in the ordination space, while a third group is composed of the localities containing individuals identified as *M. sinacacolinii*. One hundred and fifty principal components (PC) were retained which explained 88.1% of the total variance of the data. When the Northern Zone was analysed separately, *M. decastroi* and *M. mexicana* conformed three groups: the first one comprising the wild localities of *M. mexicana* (i.e., FA-1 and FA-15), the second one including cultivated and wild localities of *M. decastroi* (i.e., FA-12 and FA-13), and the third group with only the wild localities of *M. mexicana* (i.e., FA-10, FA-11, FA-9, and FA-2; Figure 3B). In this analysis 50 PCs were retained which explained 97.1% of the total variance. Similarly, when analyzing the Southern Zone separately excluding the clearly differentiated localities identified as *M. sinacacolinii*, the localities of *M. zoquepopolucae* formed a different group from *M. lopezobradorii* (Figure 4C), whereby the 5 retained PCs explained 40.3% of the total variance. The three-grouping pattern recorded in the complete dataset was retrieved when only wild localities were analysed (Figure 4D). One hundred and fifty principal components (PC) were retained, which explained 88.5% of the total variance of the data. Finally, the separation between *M. decastroi* and *M. mexicana* observed in the complete dataset remained when only the wild localities were examined (Figure 4E), in which 60 PCs retained explained 88.2% of the total variance.

AMOVA showed that the proportion of the genetic variance explained among localities of all species was 65.95%, while the genetic variance within localities was 34.05% (results not shown). When localities were grouped according to the Northern and the Southern Zone (Figure 2) the percentage of variation explained by this grouping was 22.13%. When localities were grouped according to three species groups (*M. decastroi*–*M. mexicana*, *M. lopezobradorii*–*M. zoquepopolucae*, and *M sinacacolinii*), this explained 73% of the genetic variation in the dataset. When four species groups were considered (*M. decastroi*, *M. mexicana*, *M. lopezobradorii*–*M. zoquepopolucae*, and *M. sinacacolinii*), this declined to 67.85% and in five species groups, the explained variation was 67.72%.

**Figure 5:**
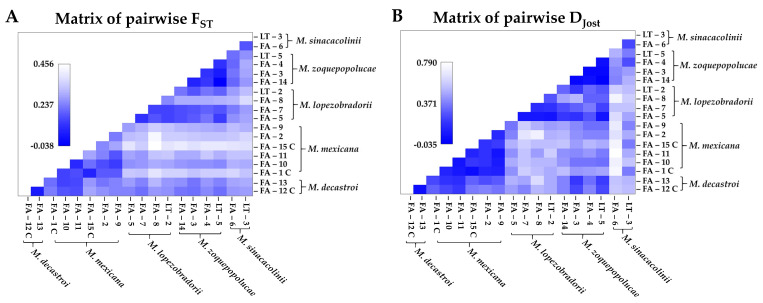
Pairwise F_ST_ and D_JOST_ values between the 18 localities of *Magnolia* species in Puebla and Veracruz, Mexico, visualized as a heatmap, C=cultivated. (**A**) Pairwise F_ST_ values. (**B**) Pairwise D_JOST_ values. Locality metadata can be found in Table 5.

Pairwise F_ST_ and D_JOST_ values between the 18 localities are tabulated in Table 2 and visualised in an accompanying heatmap in Figure 5. Pairwise F_ST_ values between localities varied between −0.04 and 046. Pairwise D_JOST_ values between localities varied between 0.00 and 0.79. The pairwise F_ST_ values and D_JOST_ for the *M. decastroi* localities, of which one is a cultivated locality (FA-12) and the other a wild one (FA-13), is 0.03 for both measures. The pairwise F_ST_ values and D_JOST_ between the wild *M. mexicana* localities (FA10, FA-11, FA-2, and FA-9) ranged between 0.06–0.22 and 0.06–0.17, respectively. The pairwise F_ST_ values and D_JOST_ between the wild *M. mexicana* localities (FA10, FA-11, FA-2, and FA-9) and the cultivated localities (FA-1 and FA-15) ranged between 0.06–0.26 and 0.02–0.20, respectively. The pairwise F_ST_ values and D_JOST_ between the *M. lopezobradorii* localities ranged between 0.08–0.22 and 0.06–0.27, respectively. The pairwise F_ST_ values and D_JOST_ between the *M. zoquepopolucae* localities ranged between −0.04 to 0.09 and −0.03 to 0.04, respectively. The pairwise F_ST_ values and D_JOST_ between the *M. sinacacolinii* localities were 0.15 and 0.23, respectively.

When F_ST_ values were further compared with each other, only the localities with sample sizes higher than 10 were considered, as the two parameters are largely affected by unequal sample size [50,51]. The pairwise comparison of FA-13 (N = 33) vs. FA-10 (N = 31), which are wild localities of the species *M. decastroi* and *M. mexicana*, stood out as this is an interspecific comparison with allelic differentiation (D_JOST_: 0.17) and fixation index (F_ST_: 0.11) in the range of intraspecific comparisons. With the exception of the FA-13 vs. FA-10 pair, the allelic differentiation (D_JOST_) showed smaller values for the intraspecific comparisons (D_JOST_: 0.02–0.18), and larger values for the interspecific comparisons (D_JOST_: 0.02–0.75). For the fixation index, there was no clear separation between intraspecific and interspecific values. The two localities of *M. decastroi* and FA-1 and FA-15 of *M. mexicana* showed little fixation (F_ST_: 0.03 and 0.06, respectively). The locality pair with the highest intraspecific pairwise F_ST_ was that of FA-15 and FA-2 of *M. mexicana* (F_ST_: 0.22). Due to small sample sizes of the *M. zoquepopolucae* localities, this species was omitted from a detailed study of the pairwise genetic differentiation at the level of localities, yet at the level of species (see further), it was included.

**Figure 6:**
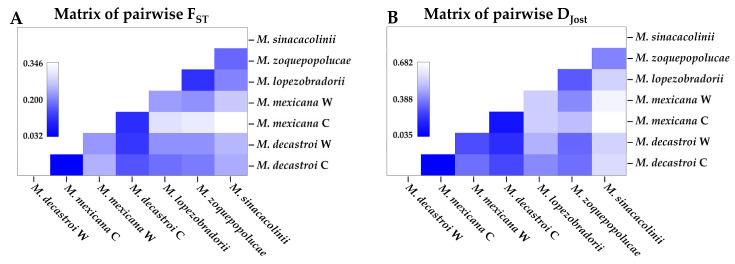
Pairwise F_ST_ and D_JOST_ values between the five *Magnolia* species studied in Puebla and Veracruz, Mexico, visualized as a heatmap. W = wild, C = cultivated. (**A**) Below the diagonal pairwise F_ST_ values are tabulated. (**B**) Pairwise D_JOST_ values are tabulated. Locality metadata can be found in Table 5.

Pairwise F_ST_ and D_JOST_ values between the five presumed species, respecting cultivated and wild populations separately, are tabulated in Table 3 and visualised in Figure 6. Pairwise F_ST_ values (excluding the cultivated populations) varied between 0.11 and 0.26. Pairwise D_JOST_ values varied between 0.19 and 0.64. The lowest pairwise differences (excluding the cultivated populations) were between the wild localities of *M. decastroi* and *M. mexicana* (F_ST_: 0.12; D_JOST_: 0. 19); and between *M. lopezobradorii* and *M. zoquepopolucae* (F_ST_: 0.11; D_JOST_: 0.28). The highest pairwise differences (excluding the cultivated populations) were between the wild *M. mexicana* localities and *M. sinacacolinii* (F_ST_: 0.26, D_JOST_: 0.64).

#### 2.1.3. Genetic Diversity

The genetic diversity data obtained are summarized per locality and per species in Table 4. The number of alleles (A) varied between 2.85 and 7.7. The allelic richness, rarified to 12 individuals (A_R_ (12)), varied between 2.69 and 6.55. The allelic richness, rarified to 24 individuals (A_R_ (24)), varied between 3.46 and 7.23. The number of private alleles (A_P_) varied between 0 and 33. Observed heterozygosity (H_O_) varied between 0.45 and 0.72. Expected heterozygosity (H_E_) varied between 0.41 and 0.69.

When respecting the current species delimitations, we consequently saw the trend of lowest genetic diversity for localities identified as *M. mexicana* and the highest genetic diversity for localities identified as *M. sinacacolinii*, with the exception of the parameter P. *Magnolia sinacacolinii* had a very high number of private alleles (41) compared to the other species, for which A_P_ ranged from 0 to 12. Significant inbreeding was detected for FA-12 (*M. decastroi*), LT-2 (*M. lopezobradorii*), FA-10 (*M. mexicana*), and for both localities of *M. sinacacolinii*.

Comparing the genetic diversity between the localities, FA-15 had the lowest genetic diversity values. FA-1, FA-2, FA-10 (all *M. mexicana*), and LT-2 (*M. lopezobradorii*) had similar A_R_(12) values (A_R_(12) = 3–4) and localities FA-7 (*M. lopezobradorii*), FA-12 and FA-13 (both *M. decastroi*) had higher A_R_ (12) values (A_R_(12) = 4.5–6) and the FA-6 (*M. sinacacolinii*) showed the highest number (A_R_(12) = 6.5). Regarding private alleles, FA-6 (*M. sinacacolinii*) had the highest number (A_P_ = 33) and FA-7 the second highest (*M. lopezobradorii*, A_P_ = 4). Five out of the 18 localities showed significant signs of inbreeding (Table 4). Locality FA-6 was the locality with the highest, significant inbreeding coefficient (F_IS_ = 0.19).

### 2.2. Assessment of Conservation Status

Based on our data, *M. sinacacolinii* and *M. zoquepopolucae* were assessed as Endangered (EN) and these assessments in the meantime have been published [52,53]. For both species, the data revealed that their current population trend is decreasing, and the main threats were habitat destruction, fragmentation of ecosystems, and extensive change in land use; especially shifting agriculture practices that are widespread among the local people, as well as selective logging along with conversion of forest for pasture (cattle ranching) and construction of transportation/service corridors. Area of occupancy (AOO) and extent of occurrence (EOO) both showed a continuing decline. In terms of diseases, symptoms resembling mosaic virus disease were observed on the leaves of some juvenile individuals in San Andrés Tuxtla and Soteapan (both municipalities are located at extremes of the distribution) of *M. zoquepopolucae* (pers. obs.).

## 3. Discussion

### 3.1. Disentangling the Species

Speciation is a continuous process whereby two separately metapopulation lineages acquire more differences or evidence, either morphological, (phylo)genetic, or other lines of evidence [54]. Using SSR data, we gathered molecular evidence to discuss where exactly in the continuum between populations and species our studied *Magnolia* individuals at the collection localities are found. If there is no gene flow occurring anymore between two localities for ample amount of evolutionary time, their populations will become more and more genetically differentiated over time [48,54]. However, a few migrants between such populations can reset such genetic differentiation, hence maintaining the concept of a metapopulation lineage or species [55,56].

Our Structure analysis on the complete dataset (Figure 3A–C) put forward the separation between the North and South, which is supported by the geography of the study area. The Northern Zone corresponds with the Sierra Madre Oriental and the Huasteca climate zone, characterized by alkaline soils, whereas the Southern ones is located in the isolated volcanic mountain range of Los Tuxtlas with extremely humid climate, characterized by neutral soils, and surrounded by the Gulf coast plain with acid soils. The DAPC plot (Figure 4A,D) supported this pattern: along the primary, horizontal axis (ignoring the secondary, vertical axis), we observe indeed a gap between the Northern and Southern samples. Based on this first Structure result, one could argue that we thus have two main groups or metapopulations and hence two species. As we expected K to be 4 or 5 according to the described species, we observe in Figure 3D and 2E that this genetic structure is clouded by a strong genetic signal splitting of the *M. mexicana* localities into two genetic clusters, following a split between the cultivated localities FA-1 and FA-15 (Table 5) and the wild localities FA-10, FA-11, FA-9, and FA-2. Interestingly, when only analysing the wild localities (Figure 3F–H): excluding sampling localities FA-1, FA-15, and FA-12 (Table 5), the Structure analysis does find five genetic clusters with high confidence, corresponding to the five described species.

Next to the Structure result, the other analyses and parameters put forward the recognition of *M. sinacacolinii* as a separate species within the Southern Zone. Firstly, we saw in the DAPC plots (Figure 4A, D) that the secondary, vertical axis, which also had a significant contribution to the discrimination of the genetic clusters (evidenced by the DA values) clearly separates *M. sinacacolinii* from the cluster that exists of individuals belonging to *M. lopezobradorii* and *M. zoquepopolucae*. The potential null alleles (Table 1) and/or high inbreeding (Table 4) for *M. sinacacolinii* could explain the result of the Structure analysis on the complete dataset not detecting this species as a “significantly” different genetic cluster (Figure 3A), while in the DAPC analysis (Figure 4) this pattern was very clear, as Structure analyses are aimed to find random mating genetic clusters [57]. Secondly, the AMOVA test showed the highest percentage of variation explained by the grouping of the localities according to three groups (73%): *M. decastroi*–*M. mexicana*, *M. lopezobradorii*–*M. zoquepopolucae*, and *M sinacacolinii*, compared to grouping according to the Northern and the Southern Zone (22.13%). Lastly, the remarkably high number of private alleles (Table 4) highlights this species as being a very distinct entity. Our genetic results are strengthened by morphological and ecological data. Morphologically *M. sinacacolinii* is easily discriminated from the other four (alleged) species by tree architecture, leaf texture and pubescence and fruit morphology [47], which is very remarkable given the close geographic proximity of the species to *M. zoquepopolucae* and *M. lopezobradorii*. Ecologically *M. sinacacolinii* only grows within the Los Tuxtlas area at lower elevations in localities protected from the northern winds, in contrast with *M. lopezobradorii* and *M. zoquepopolucae* that occur at much higher elevations, both in localities protected from and exposed to these northern winds. Hence, based on genetic, morphological, and ecological evidence we state that there are (at least) three species in our dataset: one in the Northern Zone, and two in the Southern Zone.

Within the Northern Zone, the sample localities FA-12 and FA-13 identified as *M. decastroi* are a distinct genetic cluster (Figure 3D,E,H), but here the interspecific genetic differentiation (Table 3; Figure 6) between these localities and the other northern localities is less pronounced (F_ST_: 0.12, D_JOST_: 0.19) and in the range of the intraspecific genetic differentiation (Table 2; Figure 5). This result is even more striking when taking into consideration that the northernmost wild locality of *M. mexicana* (FA-10) and the most southernly located *M. decastroi* wild locality (FA-13) are the interspecific locality pair in the range of the other intraspecific values (Table 2, Figure 5). Hence, based on our SSR data FA-13 (and FA-12) would be considered a separate population from the other *M. mexicana* populations instead of a separate species. Morphological and physiogeographic data are not in accordance with this result. There is one morphological characteristic that distinguishes *M. decastroi* and *M. mexicana*: the pubescence of the flower bracts [47], which is difficult to observe because the flower bracts are deciduous. During the sampling it was first assumed that FA-12 and FA-13 were the southernmost localities of *M. mexicana*, and only after the SSR results showed that this particular population consistently was a separate genetic cluster (Figure 3) the pubescence was detected on three individuals in the field from which herbarium vouchers were collected. Physiogeographically, within the Northern Zone, the Sierra de Zongolica that holds the populations FA-12 and FA-13 corresponds to the Southern portion of the Sierra Madre Oriental that is isolated from the northernmost *Magnolia* localities by the Trans-Mexican Volcanic Belt and a much more humid climate. As the data are somewhat conflicting, we recommend that for a more final decision on the recognition of the species it would be necessary to genetically compare populations or individuals from the type locality of *M. decastroi* (slightly more to the South, in the Sierra Mazateca, Oaxaca, around 75 km southwards of the sampled localities), as well as other recently found localities in the same region with FA-12 and FA-13. Moreover, there might be more undocumented localities, hence, more explorations can provide more insight in gene flow between localities that are identified as *M. mexicana* and *M. decastroi*. Taken all together, based on the data gathered so far, it can be concluded that the studied wild FA-13 and cultivated FA-12 will most likely be synonymized with *M. mexicana*.

Similarly, the Southern Zone localities FA-3, FA-4, FA-14, and LT-5 identified as *M. zoquepopolucae* had very little genetic support for being treated as a separate species (Table 2, Figure 4) compared to the localities FA-5, FA-7, FA-8, and LT-2 identified as *M. lopezobradorii*. Firstly, in the Structure results of the complete dataset the two species are not retrieved in the K = 4 replicates (Figure 3D), and only retrieved in two of the ten replicates in the K = 5 cluster (Figure 3E). However, in the dataset comprising only the wild individuals the two species are found as two genetic clusters *cf.* the species (Figure 3H). Secondly, the pairwise genetic fixation between the species (F_ST_: 0.11: Table 3; Figure 5) is in the lower range of that found in intraspecific comparisons (F_ST_: 0.03–0.22: Table 2; Figure 4) and the pairwise allelic differentiation (D_JOST_: 0.28) is in the range of the wild *M. decastroi*–*M. mexicana* pairwise comparison (D_JOST_: 0.17&0.25) rather than the pairwise comparisons which we are positive to be interspecific (D_JOST_: 0.46–0.75). These genetic results are confirmed by the absence of a significant morphological distinction found by [47]. The only argument left in favour to discriminate the two described species is the geography: the species are around 55 km apart and are located on different volcanoes. Taken all together, based on the data gathered so far, we conclude that the studied populations can be considered to be two genetically differentiating populations of the same species: *M. zoquepopolucae*.

Comparing the found measures of genetic differentiation of the two species complexes under consideration of being over-splitted (i.e., *M. decastroi*–*M. mexicana* F_ST_: 0.12; D_JOST_: 0.19 and *M. lopezobradorii*–*M. zoquepopolucae* F_ST_: 0.11; D_JOST_: 0.28) with other studies of Neotropical *Magnolia* populations, we found that the F_ST_ values were lower, or well within the lower half of what is considered intraspecific genetic differentiation. For example, in [21] *M. pedrazae* and *M. schiedeana* were reconsidered to be one species with F_ST_ values between the populations ranging between 0.053 and 0.283. In the study on Caribbean Magnolias of [42], intraspecific F_ST_ values ranged between 0.044 and 0.223. In [58], *M. nuevoleonensis* and *M. alejandrae* were proposed to be synonymised with *M. dealbata* with F_ST_ values that ranged between 0.21 and 0.43.

In the debate of species delineation, both in our study and in other SSR studies to date, we must acknowledge that, although the SSR markers are valuable in studying the stochastic processes shaping the populations’ genetic diversity, it is only partial evidence. Genes and their adaptation to a specific environment can be what differentiates one species from another, while the structure patterns in neutral DNA still lags behind [21,22,42].

### 3.2. Patterns of Gene Flow Between, and Inbreeding within the Wild Sample Localities

Overall, we observed great variation in genetic differentiation among localities within the alleged five species (Figure 5 and Figure 6, Table 2 and Table 3), whereby the (wild) populations of *M. mexicana*, *M. lopezobradorii* and *M. sinacacolinii* showed levels of genetic differentiation of moderate and great genetic differentiation [59] for both the amount of genetic fixation and the amount of allelic differentiation. This means that the past and current gene flow among the sampled localities overall is low. As our current sampling comprised both adults and juveniles, we expect that in the fragmented landscape this result of overall genetic differentiation will become only increasingly pronounced if there are no conservation management actions to reverse this differentiation [22,60].

As gene flow between the populations appears to be limited, more inbreeding is expected. However, significant inbreeding in wild localities was detected only in the wild localities LT-2 (*M. lopezobradorii*), FA-10 (*M. mexicana*), and in the two *M. sinacacolinii* populations (FA-6 and LT-3) (Table 4). In all the other localities there was no signal for inbreeding at hand. This pattern of limited gene flow and little inbreeding is similar to the study of [42] and can be attributed to the evolutionary resilience of the tree habit of strongly overlapping generations [61] and potentially the reproductive biology of the species promoting outcrossing [62]. Only the localities of *M. sinacacolinii* probably have reached a threshold of a too small population size, for the populations to remain genetically resilient to inbreeding.

It is preferable for plants to maintain high levels of genetic variation within their populations, as their sessile nature can lead to the evolution of locally adapted ecotypes [63]. However, in many woody plant and tropical tree species, high levels of genetic variation are reported to be found within populations [49,64,65,66], while a small fraction of diversity is observed between populations. On the other hand, species with a wide distribution range have greater genetic variability within populations than species with a more restricted distribution [66,67].

### 3.3. Defining the Conservation Units of the Magnolias of Veracruz

Conservation units, also called management units [68], can either be populations within a species or can even be synonymised with the species as a whole [69,70]. Based on our data we recommend recognising maximally five conservation units *cf*. the genetic clusters retrieved by the Structure analysis on the wild localities (Figure 3H). Each of these genetic clusters represents a collection of localities currently identified as one described species (Figure 3). We recommend to enhance gene flow among the different sample localities within the five genetic clusters (Table 4) and treat each described species as one conservation unit, not divided further in separate managed localities or populations. This because of various reasons: (1) the intensive sampling executed for this study retrieved a low number (N < 10) of *Magnolia* trees at 9 of the 15 sampled wild localities (Table 4); (2) between the localities within the five genetic clusters there is up to great intraspecific genetic differentiation (Figure 5, Table 2); and (3) in 4 out of the 15 sampled wild localities there is significant inbreeding detected (Table 4).

Although geographically distinct and at one point described as two species [26,27], we recommend to recognise only four conservation units. This by managing the eight localities (Table 5) that are now identified to contain individuals of *M. lopezobradorii* and *M. zoquepopolucae* as one. Although the data do clearly support them to be two genetically differentiated populations that are not randomly mating (Figure 3F–H), the collected demographic data of the sampled localities is too precarious (Table 4 and Table 5). We recommend translocating between the two populations because the 15 individuals at the four localities identified as *M. zoquepopolucae* are a relict population of which most are adults (i.e., there is no recruitment) (Table 4). Chances of finding more individuals and/or localities of this genetic cluster are very low (as opposed to the *M. decastroi* genetic cluster, see next paragraph).

Lastly, we recommend further investigation to consider managing the wild population FA-12 identified as *M. decastroi* together with the populations of *M. mexicana*, i.e., the other sampled populations in the Northern Zone (Figure 2). We recommend an additional molecular (conservation genetic, or phylogenetic) study that expands the sampling and includes the type population of *M. decastroi*. In the meanwhile, the wild population FA-13 containing 33 individuals with no significant inbreeding (Table 4) could best be managed separately, as one conservation unit.

### 3.4. Priorities for Magnolia Conservation in Veracruz

We notice that, overall, *M. mexicana* had the lowest genetic diversity, while *M. sinacacolinii* was the most genetically diverse (Table 4). Interestingly, when taking into account the IUCN status of the species, *M. mexicana* is denoted as VU (Vulnerable), while the other three species (i.e., *M. decastroi*, *M. zoquepopolucae*, and *M. sinacacolinii*) are EN (Endangered) (*M. lopezobradorii* is DD). This illustrates that even though the number of individuals and species is (estimated to be) larger—which are the main parameters for calculating the IUCN Red List status [31,71], the genetic diversity of those species might actually be more alarming, likely due to a century-long collection of flowers for medicinal uses [72].

The interesting pattern observed in the *M. sinacacolinii* FA-6 locality, i.e., high genetic diversity, high number of private alleles, but inbreeding (Table 4), could be due to the population structure, where the adult individuals still harbour much genetic diversity, significantly different from the other studies species (i.e., private alleles); yet, recently a reproductive event of a few more related individuals, or perhaps even geitonogamy, delivered that this genetic variation that is found more in a homozygotic state.

It is hard to state which has priority for conservation as each of the four conservation units have a different set of challenges ahead, which threaten their existence. However, out of the four proposed conservation units, *M. sinacacolinii* is flagged the most, as this species has strong inbreeding detected in both populations and only two (known) localities of which one only has four (known) individuals (Table 4).

### 3.5. Ex Situ Collections Versus In Situ Populations

The Structure result (Figure 3C,E) was striking, as the division among the *M. mexicana* individuals in two genetic clusters was unexpected. FA-15 is a completely cultivated locality, introduced through the Francisco Javier Clavijero Botanical Garden of the Instituto de Ecología, A.C. (JBFJC). It appears that genetically, FA-1 is a mixture of the wild individuals and the FA-15 individuals. Villagers in the area commented that *M. mexicana* used to be abundant, but its population size has been reduced mainly due to northern winds. The JBFJC data (pers. comm.) indicate that the arboretum individuals were brought from Northern Veracruz, near locality FA-9, which could correspond to extinct populations. This could be confirmed during our field work, as many localities from where individuals of *M. mexicana* had been recorded according to herbarium vouchers, were no longer found, due to deforestation and coffee plantations.

For *M. mexicana*, the allelic diversity [A_R_ (12)] in the localities that consist of ex situ individuals (FA-1 and FA-15) is lower than compared to the wild populations (Table 4), although the difference is not that pronounced. This stresses the importance of sampling a good variety of mother trees to capture the genetic diversity of the population [61,70]. This is exemplified by the *M. decastroi ex situ* collection FA-12 compared to FA-13: here the allelic richness of the ex situ collection is higher than the in situ (sampled) population with two private alleles (A_P_). However, for the FA-12 population, the inbreeding coefficient (F_IS_) was significant, likely caused by more kinship among the ex situ population.

### 3.6. Implications for Conservation

Based on the genetic data, we now define three species with certainty and updated their IUCN Red List statuses of two of them accordingly. The importance of adhering to the Red List guidelines lies in the fact that it is the world’s leading instrument of its kind. It provides alerts on the state of the world’s biodiversity; its applications at the national level enable decision-makers to consider the best options for the conservation of species [31]. The current IUCN Red List assessments still respect the five species delimitation based on the species descriptions.

We propose a preliminary conservation strategy for the four proposed conservation units, based on three main guidelines: diffusion, protection, and propagation. We urge that for efficient conservation, local people are included to achieve an integrated strategy so that they become decision-makers and are involved in the preservation of endangered plant species [73,74]. The first guideline: diffusion is aimed to ensure that knowledge of the species reaches more inhabitants and local organizations in the areas where they are naturally distributed, for example, through information posters and talks to local people. The second guideline: protection aims at ensuring that out of the currently known individuals, no further trees are lost. The third guideline: propagation aims at increasing the genetic diversity and number of individuals at localities. For this purpose, a method of manual propagation by seed has already been developed for species of the *Talauma* section from Cuba that has worked for other *Magnolia* species in various Latin American countries, and agreements have been made with various organizations that also have experience cultivating Magnolias in Mexico [75,76]. It is important that these three guidelines are carried out together and are seen as a process, although depending on each species or conservation unit, it may be necessary to place more emphasis on one than the other. However, in general, it can be stated that the most important actions are diffusion and protection, protecting what is known to remain, while trying to inform the local communities.

Given that there is still adequate genetic diversity present (Table 4), it is proposed to propagate the studied species both in situ and ex situ, which are contemplated in different protection strategies, such as the botanical garden conservation strategy [77], as well as in the Mexican strategy for plant conservation [73]. For the inclusion of species in ex situ collections, arboreta in national botanical gardens can be considered. This is currently executed at the JBFJC which has already successfully propagated other plant groups [78,79,80]. Because the genetic diversity within the three conservation units appears to be limited by gene flow (Figure 5 and Figure 6, Table 3 and Table 4), we propose that translocations between localities can be executed and, preferably, that their outcome is monitored. Although we risk undoing local adaptation and outbreeding [81,82] as we only quantified the populations with neutral genetic data [42], the genetic consequences of fragmentation and subsequent loss of genetic diversity are far greater [70]. As a matter of urgency, two actions are proposed in the Southern Zone: collecting seeds from the small populations and add them to the large populations, while focusing on protecting these larger populations. In the Northern Zone, we suggest focusing on the small populations and reforest them from the other populations to make them larger again. Although we currently only propose to translocate seeds between localities or populations, future work should consider connecting the forest fragments in the landscape, so that gene flow within the conservation units occurs naturally.

Finally, it is proposed to apply all of the above strategies to *M. wendtii* (including genetic evaluations, taxonomy, and conservation), the only species from Veracruz in the section *Talauma* that could not be included in this study. More exploration work is needed, primarily in the border area of Oaxaca and Veracruz, as only a small population has so far been identified in the latter state, but the rainforest in between these two states is extremely fragmented.

## 4. Materials and Methods

### 4.1. Study Species, Study Zones, and Sampling

Five species of *Magnolia* sect. *Talauma* were studied (Figure 1), of which four were recently described [26,28] and segregated from *M. mexicana*, namely, *M. decastroi*, *M. lopezobradorii*, *M. sinacacolinii*, and *M. zoquepopolucae*. The only species belonging to this section in Veracruz that was not included was *M. wendtii* from southern Veracruz. *Magnolia wendtii* is only known from an area with an extremely high deforestation degree, and the sample number was too small to include.

Two zones were considered according to the natural distribution of the species: the Northern Zone which corresponds to a part of the Sierra Madre Oriental [83,84,85] in the states of Puebla and Veracruz, encompassing five natural regions; in contrast, the Southern Zone comprises the natural regions of Los Tuxtlas and Olmeca [11] in Veracruz (Figure 2). Nine field trips were conducted between February 2016 and January 2020, three of them in areas unexplored for *Magnolia* (especially around the municipalities of Xalapa and Coatepec in Veracruz). The entire distribution area of the five species was covered, visiting two states, 12 municipalities, and 31 localities (Table 5), covering areas without previous records for *Magnolia* (central Veracruz) and others that had not been visited since the 1980s (northern Puebla and southern Veracruz).

A total of 254 young leaf samples were collected for molecular analyses (approximately 5 cm^2^ of the leaf avoiding veins) and dried in silica gel. These belonged to 18 localities, of which three localities contained cultivated individuals. A first cultivated locality, FA-15 was in situ (private home in Coatepec). The second cultivated locality, FA-1 was in situ (public parks in Xalapa) and the third cultivated locality was ex situ (greenhouse of the Instituto Tecnológico Superior de Zongolica, ITSZ). The first two are identified as *M. mexicana* and the third as *M. decastroi* (Table 5). To correctly identify the individuals at the localities, 145 herbarium vouchers were collected, representing at least one individual at each sample locality (55 collection numbers with their respective duplicates), which will be deposited in the herbaria of the Instituto de Ecología, A.C., Centro Regional del Bajío (IEB), National herbarium of Mexico (MEXU), and Instituto de Ecología, A.C. (XAL).

The aim was to sample 30 individuals from each locality, and when this was not possible, all individuals were collected. In case the number of individuals exceeded 30, individuals were selected randomly covering the whole area. In each locality, tree height, GPS coordinates, habitat description, phenology (if the tree was flowering or fruiting), age class (adult or juvenile, based on whether it had reproductive organs or scars left by them), and DBH (diameter at breast height) were recorded for each individual. In total, 121 individuals were classified as adults and 157 as juveniles.

In order to classify the sampled localities according to the described *Magnolia* species (Table 5) and to obtain a complete overview of the morphological variation of the species involved, 136 herbarium vouchers have been studied. The following herbaria in the states of Puebla and Veracruz were visited: Centro de Investigaciones Tropicales, CHAPA, CIB, CORU, ENCB, FCME, FEZA, HUAP, IEB, IZTA, Estación de Biología Tropical Los Tuxtlas, MEXU, QCA, UAMIZ, XAL, XALU and ZON; complemented by a study of digitally available collections in F and MA (acronyms are according to [86]). Photographs were taken of all specimens and loans were requested from each of these herbaria. The detailed visual evaluation of the phenotypic traits of these specimens, as well as our own collections, have resulted in a list of 35 characters to distinguish the species [47]. Moreover, since the protologue of the recently described species was mainly based on differences in the number of carpels, this feature has been statistically analysed by [47].

### 4.2. DNA Extraction and PCR

DNA extraction was performed using the CTAB method modified by [87]. A total of 181 existing microsatellites created from *M. cubensis*, *M. dealbata*, *M. lacandonica*, and *M. mayae* were evaluated [42]. DNA quality was assessed using a spectrophotometer NanoDrop 1000 Spectrophotometer (Thermo Fisher Scientific, Waltham, MA, USA). Forward primers were linked to a universal strand to achieve multiplex pooling. The universal tags used (T3, M13, Hill, and Neo) were those recommended by [42].

PCR reactions were prepared under the following conditions: denaturation at 95 °C for 15 min followed by 35 cycles of denaturation at 94 °C for 30 s, annealing at 57 °C for 1.30 min, extension at 72 °C for 1.30 min and final extension at 72 °C for 10 min, extension at 72 °C for 1.30 min and final extension at 72 °C for 10 min. Each Master Mix used for the reaction contained: 0.2 μM forward primer, 0.2 μM reverse primer, DNA (diluted in 1 × TE buffer) and QIAGEN Multiplex PCR Kit. The total PCR volume was 5 μL, of which 1 μL was diluted DNA (1/10), 2 μL of Qia Multiplex PCR master Mix, and 2 μL of primer mix (forward and reverse primers). When testing SSR primers for amplification of a single PCR product, the PCR products were run on 1% agarose gel for 1 h at 115 V and 400 mA. Subsequently, the gel was stained in ethidium bromide for 25 min, placed under UV light and the digital image was captured. Of the SSR primers delivering a single product, fragment analyses were performed by ABI 3130XL fragment analyser (Thermo Fisher Scientific, Waltham, MA, USA) using the GeneScanTM 500 LIZ^TM^ (Thermo Fisher Scientific, Waltham, MA, USA) as a ladder in “singleplexes” and after verification, de novo designed multiplexes. The products were genotyped in Geneious v. 8.1.9 [88].

### 4.3. Genetic Analysis and Characterisation

The software Convert v. 1 [89], Create v. 1.38 [90] and PGDSpider v. 2.1.1.5 [91] were used to convert both data sets to the different formats used by the other programs mentioned in the following sections.

#### 4.3.1. Null Alleles and Linkage Disequilibrium

Null allele detection was carried out using Microchecker v. 2.2.3 [92], setting the maximum expected size of the allele: 400, confidence interval: 95%, 1000×, not including the alleles with a zero value. To calculate the frequencies of the potential null-alleles we used ML-Null Freq v.1 [93] with 1 000 randomisations.

The linkage disequilibrium (LD) was tested by exact probability test using Genepop v. 4.3 [94,95] applying the following parameters: number of dememorization steps: 10,000, number of batches: 1000, iterations per batch: 50,000; sequential Bonferroni correction was applied to correct the nominal *p*-value of 0.05 for multiple testing [96].

#### 4.3.2. Genetic Structure

Genetic structure analyses were carried out using Structure v. 2.3.4 [97,98]. We decided to use two datasets. In the first one, called the complete dataset, all sampled individuals (both wild and cultivated) were considered. In the second one, only the individuals of the 15 localities with exclusively wild individuals were maintained (Table 4). For both datasets, the number of genetic groups K was set to run from 1 to 30, with 10 replicates each. The upper bound of K = 30 was chosen to allow for substructure within the 15 or 18 sample localities. Each run was performed using 100,000 iterations as burn-in and 100,000 repetitions of the Markov chain Monte Carlo (MCMC) after the burn-in. The ancestry model was the admixture model. The allele frequency model was set to allele frequencies independent, as we expected there to be different species in the dataset, which have been separated for a substantial amount of evolutionary time. After the complete dataset was run, we repeated the Structure analysis for the two main obtained genetic clusters (GC) to further investigate substructure. For these analyses we used the same parameter settings, except that the upper limit of K was set to be twice the number of sampling locations corresponding to each GC obtained, and the allele frequencies set to be correlated. We determined the optimal K of each of the eight structure runs, using the online resource of Structure Harvester [99] whereby we examined the ΔK plots [57] and the mean likelihood plots. Bar plots were visualised using DISTRUCT v. 1.1 [100].

A discriminant principal component analysis (DAPC) in R [101] using the adegenet R package [102] was carried out to further investigate the number and relationship of the genetic clusters following the method proposed by [103] and the recommendation of [104]. For both datasets, 150 Principal Components (PCs) were retained. The number of PCs to retain for the eigenvalues of the principal component analysis (PCA) was determined using cross validation.

Analyses of Molecular Variance (AMOVA) were performed, defining different groups. Firstly, we performed an AMOVA on all the individuals, not defining any groups. Next, AMOVA was run dividing the populations into two, three, four or five groups, according to the Structure and DAPC results and discussion on the number of true species. Significance of AMOVA components was tested with 1 000 permutations in Arlequin v. 3.5.2.2 [105].

To quantify the genetic differentiation among the localities and among the genetic clusters, we ran two analyses using the diveRsity R package [106]. One analysis was run respecting the localities (i.e., 18 “populations”) and one was run respecting the five species and separating cultivated and wild localities in *M. decastroi* and *M. mexicana*. Pairwise F_ST_ [50] and D_JOST_ [51] were calculated using 1000 bootstrap replicates.

#### 4.3.3. Genetic Diversity

Allele richness (A_R_), number of alleles (A) and inbreeding coefficient (F_IS_) were calculated in FSTAT v. 2.9.3.2 [107]; expected (H_E_) and observed heterozygosity (H_O_), population polymorphism (P), and private alleles (A_P_) were evaluated in GenAlEx v. 6.5 extension [108] for Microsoft Excel; and deviations from Hardy–Weinberg equilibrium (HWE) were tested in Genepop v. 4.3 [94] with the following parameters: number of dememorization steps: 10,000, number of batches: 200, iterations per batch: 50,000.

### 4.4. Assessment of Conservation Status

IUCN Red List categories and criteria [71] were applied to define the conservation status of the resulting species (taxonomic changes were not yet formalized [47]). Comments from local people regarding the increase or decrease of individuals were considered, as well as using herbarium records used to search for individuals. Area of Occupancy (AOO) and Extent of Occurrence (EOO) were calculated in GeoCAT [109]. Threats observed in the habitats of each species were also detailed following the IUCN classification scheme [110]. All data collected on distribution, population, use, threats, conservation, etc., were captured in the IUCN Species Information Service (SIS) database to generate the final assessments.

## 5. Conclusions

In conclusion, we find genetic support for at least three out of the five studied described species, and we propose four main conservation units. The genetic evidence indicates over-splitting is most likely at hand and we recommend a formal taxonomic revision of the species, with emphasis on the *M. decastroi*–*M. mexicana* complex and the *M. lopezobradorii*–*M. zoquepopolucae* complex. Localities are exhibiting variable, case-specific levels of genetic differentiation, yet most can be classified as moderate or great, which indicates low (past) gene flow. Five of the 18 studied localities showed genetic signatures of inbreeding. The 13 populations with no signs of inbreeding indicate that random mating was maintained within the majority of populations. *Magnolia sinacacolinii* was flagged as the highest priority conservation unit, given that the species had signs of inbreeding in both its populations and a low number of known localities and individuals. However, the other three conservation units are also in need of urgent conservation management: the *M. mexicana* and *M. decastroi* conservation units had the highest intraspecific genetic differentiation reported and lowest genetic variability and the *M. zoquepopolucae*–*M. lopezobradorii* conservation unit have 6/8 relict localities that are not exhibiting gene flow between the two sampled volcanoes in Los Tuxtlas. We recommend to genetically characterise more populations of *M. decastroi* to make further tailored decisions on their conservation management. The three evaluated ex situ collections maintain a moderate to good representation of the in situ genetic diversity. The (partly updated) IUCN Red List status for the five studied species are the following: *M. decastroi*, *M. sinacacolinii*, and *M. zoquepopolucae*: Endangered (EN); *M. mexicana*: Vulnerable (VU); *M. lopezobradorii*: Data Deficient (DD). *M. wendtii* is still assessed as Critically Endangered (CR) and we were only able to find a few individuals, hence it is necessary to implement immediate in situ and ex situ conservation actions.

The studied *Magnolia* sect. *Talauma* species of Veracruz and Puebla are hereby put forward as flagship and umbrella species for conservation in the region. In this research, valuable localities were genetically quantified, which can guide conservation management, such as choice of mother trees for collection of seeds for both in situ reforestation by translocations and establishing and genetically enriching ex situ collections. It is proposed to implement a conservation strategy based on three guidelines (diffusion, protection, and propagation) in conjunction with local people, and public and private institutions. The information generated about the genetic diversity of the localities will allow guided reforestation of these species so that the survival of new localities is not affected by low genetic diversity.

## Figures and Tables

**Table 1:** Results of MICRO-CHECKER and ML-NullFreq analyses for null alleles on the Single Sequence Repeat (SSR) dataset of five *Magnolia* species in Puebla and Veracruz, Mexico. Only SSR loci for which potential null alleles were detected by MICROCHECKER are tabulated. N: sample size. NA: not available due to small sample size. Where a potential null allele was found the frequency as calculated by ML-NullFreq is given between brackets. Metadata for the locality abbreviations can be found in Table 5. Metadata of the SSR markers are specified in [42]. (C) marks cultivated sample localities as opposed to the wild sample localities.

Species	Locality	N	SSR Locus				
			MA39_009	MA39_224	MA39_287	MA42_421	MA42_471
*M. decastroi*	FA-12 (C)	30	No	YES (0.122)	No	No	YES (0.061)
	FA-13	33	No	YES (0.256)	No	No	No
*M. lopezobradorii*	FA-5	3	NA	NA	NA	NA	NA
	FA-7	27	No	YES (0.249)	No	No	No
	FA-8	3	NA	NA	NA	NA	NA
	LT-2	14	No	YES (0.219)	No	No	No
*M. mexicana*	FA-1 (C)	24	No	YES (0.202)	No	No	No
	FA-2	13	No	YES (0.187)	No	No	No
	FA-9	7	No	No (0.330)	No	No	No
	FA-10	31	No	YES (0.369)	No	No	YES (0.011)
	FA-11	4	No	No (0.202)	No	No	No
	FA-15 (C)	18	No	YES (0.396)	No	No	No
*M. sinacacolinii*	FA-6	28	YES (0.263)	YES (0.339)	YES (0.348)	YES (0.339)	No
	LT-3	4	NA	NA (0.278)	NA	NA	NA
*M. zoquepopolucae*	FA-3	5	No	YES (0.286)	No	No	No
	FA-4	3	NA	NA	NA	NA	NA
	FA-14	4	No	YES (0.387)	No	No	No
	LT-5	3	NA	NA	NA	NA	NA

**Table 2:** Pairwise F_ST_ and D_JOST_ values between the 18 localities of *Magnolia* species in Puebla and Veracruz, Mexico. Above the diagonal pairwise D_JOST_ values are tabulated. Below the diagonal pairwise F_ST_ values are tabulated. Locality metadata can be found in Table 5. In blue the intraspecific values. (C) marks cultivated sampling localities as opposed to wild sampling localities.

Species		*M. decastroi*		*M. mexicana*						*M. lopezobradorii*				*M. zoquepopolucae*				*M. sinacacolinii*	
	Localities	FA-12	FA-13	FA-1	FA-10	FA-11	FA-15	FA-2	FA-9	FA-5	FA-7	FA-8	LT-2	FA-14	FA-3	FA-4	LT-5	FA-6	LT-3
*M. decastroi*	FA-12 (30) (C)	–	0.03	0.30	0.23	0.19	0.36	0.38	0.35	0.45	0.39	0.52	0.49	0.39	0.23	0.39	0.25	0.57	0.53
	FA-13 (33)	0.03	–	0.23	0.17	0.15	0.31	0.25	0.29	0.51	0.46	0.63	0.49	0.38	0.19	0.34	0.21	0.56	0.52
*M. mexicana*	FA-1 (24) (C)	0.21	0.18	–	0.12	0.08	0.02	0.12	0.11	0.43	0.58	0.53	0.49	0.53	0.43	0.45	0.47	0.75	0.37
	FA-10 (31)	0.14	0.11	0.11	–	0.06	0.18	0.16	0.08	0.48	0.56	0.51	0.52	0.44	0.35	0.35	0.34	0.72	0.56
	FA-11 (4)	0.14	0.13	0.13	0.06	–	0.13	0.17	0.06	0.42	0.55	0.45	0.59	0.41	0.41	0.44	0.36	0.79	0.49
	FA-15 (18) (C)	0.26	0.24	0.06	0.19	0.26	–	0.17	0.20	0.50	0.59	0.53	0.54	0.54	0.45	0.52	0.48	0.73	0.36
	FA-2 (13)	0.23	0.20	0.15	0.13	0.22	0.22	–	0.14	0.39	0.66	0.72	0.50	0.51	0.42	0.39	0.39	0.65	0.48
	FA-9 (7)	0.22	0.22	0.16	0.11	0.14	0.26	0.21	–	0.35	0.58	0.50	0.46	0.46	0.40	0.33	0.33	0.70	0.39
*M. lopezobradorii*	FA-5 (3)	0.21	0.25	0.32	0.24	0.27	0.38	0.30	0.26	–	0.07	0.06	0.06	0.19	0.17	0.28	0.06	0.63	0.47
	FA-7 (27)	0.18	0.22	0.31	0.24	0.25	0.33	0.31	0.28	0.08	–	0.15	0.17	0.26	0.30	0.38	0.22	0.55	0.56
	FA-8 (3)	0.24	0.29	0.39	0.27	0.31	0.46	0.43	0.33	0.14	0.12	–	0.27	0.46	0.52	0.30	0.31	0.73	0.59
	LT-2 (14)	0.23	0.25	0.34	0.27	0.32	0.38	0.33	0.31	0.12	0.11	0.22	–	0.33	0.18	0.31	0.27	0.59	0.54
*M. zoquepopolucae*	FA-14 (4)	0.20	0.23	0.34	0.24	0.28	0.40	0.34	0.29	0.14	0.13	0.28	0.19	–	0.00	0.03	-0.03	0.39	0.49
	FA-3 (5)	0.18	0.20	0.32	0.23	0.28	0.37	0.32	0.28	0.16	0.15	0.28	0.14	0.06	–	0.00	0.01	0.39	0.44
	FA-4 (3)	0.23	0.25	0.34	0.23	0.32	0.39	0.31	0.27	0.20	0.16	0.28	0.20	0.09	0.08	–	0.04	0.43	0.25
	LT-5 (3)	0.18	0.21	0.33	0.22	0.28	0.39	0.31	0.26	0.10	0.14	0.28	0.17	-0.04	0.04	0.09	–	0.50	0.37
*M. sinacacolinii*	FA-6 (28)	0.25	0.26	0.35	0.28	0.32	0.38	0.32	0.31	0.24	0.21	0.29	0.25	0.17	0.20	0.17	0.19	–	0.23
	LT-3 (4)	0.25	0.28	0.31	0.27	0.31	0.38	0.35	0.28	0.26	0.25	0.36	0.30	0.25	0.26	0.27	0.24	0.15	–

**Table 3:** Pairwise F_ST_ and D_JOST_ values between the five *Magnolia* species studied in Puebla and Veracruz, Mexico. Above the diagonal pairwise D_JOST_ values are tabulated. Below the diagonal pairwise F_ST_ values are tabulated. Metadata can be found in Table 5; (C) marks cultivated sampling localities as opposed to wild (W) sampling localities.

Species	*M. decastroi* (C)	*M. decastroi* (W)	*M. mexicana* (C)	*M. mexicana* (W)	*M. lopezobradorii*	*M. zoquepopolucae*	*M. sinacacolinii*
*M. decastroi* (C)	–	0.04	0.32	0.25	0.38	0.33	0.55
*M. decastroi* (W)	0.03	–	0.26	0.19	0.45	0.32	0.54
*M. mexicana* (C)	0.23	0.21	–	0.1	0.52	0.49	0.68
*M. mexicana* (W)	0.15	0.12	0.1	–	0.51	0.38	0.64
*M. lopezobradorii*	0.18	0.20	0.29	0.2	–	0.28	0.54
*M. zoquepopolucae*	0.19	0.21	0.31	0.21	0.11	–	0.36
*M. sinacacolinii*	0.23	0.25	0.35	0.26	0.19	0.17	–

**Table 4:** Genetic diversity measures estimated per sampling locality of different *Magnolia* species in Puebla and Veracruz, Mexico. N: sample size; C: number of cultivated individuals; N_G_: (mean) number of genotyped individuals. A: (mean) number of alleles. A_R_: allelic richness whereby the number of individuals to which the rarefaction is undertaken is between brackets. A_P_: (mean) number of private alleles. H_O_: (mean) observed heterozygosity. H_E_: (mean) expected heterozygosity. F_IS_: inbreeding coefficient. Significant deviations from Hardy–Weinberg Proportions (HWP): * (*p* = 0.05) are only calculated at the level of locality.

Species	Locality	N	Adults	C	N_G_	P	A	A_R_(12)	A_R_(24)	A_R_ (14)	A_P_	H_O_	H_E_	F_IS_
*M. decastroi*	TOTAL	63	10	30	63	100%	5.7	NA	NA	4.85	8	0.67	0.67	0.01
	FA-12	30	0	30	30	100%	5.62	4.81	5.16	NA	2	0.66	0.67	0.03 *
	FA-13	33	10	0	33	100%	5.08	4.54	4.78	NA	1	0.68	0.64	−0.04
*M. lopezobradorii*	TOTAL	47	19	0	47	100%	7.54	NA	NA	5.95	12	0.65	0.71	0.09
	FA-5	3	2	0	3	100%	3.08	NA	NA	NA	0	0.62	0.5	0.08
	FA-7	27	3	0	27	100%	6.7	5.7	6.34	NA	4	0.70	0.69	0.00
	FA-8	3	3	0	3	92.31%	2.85	NA	NA	NA	2	0.67	0.49	−0.17
	LT-2	14	11	0	14	100%	4.15	4.09	NA	NA	0	0.56	0.59	0.09 *
*M. mexicana*	TOTAL	97	42	27	97	100%	5.62	NA	NA	4.57	5	0.52	0.60	0.14
	FA-2	13	5	0	13	100%	3.46	3.46	NA	NA	2	0.47	0.46	0.01
	FA-9	7	6	0	7	92.31%	2.92	NA	NA	NA	0	0.50	0.49	0.06
	FA-10	31	10	0	31	100%	4.46	4.13	4.32	NA	1	0.61	0.62	0.04 *
	FA-11	4	3	0	4	100%	3.00	NA	NA	NA	0	0.58	0.50	−0.01
	FA-1	24	10	8	24	92.31%	3.54	3.3	3.46	NA	0	0.47	0.49	0.06
	FA-15	18	8	18	18	84.62%	2.85	2.69	NA	NA	0	0.45	0.41	−0.09
*M. sinacacolinii*	TOTAL	32	16	0	30.77	92.31%	8.69	NA	NA	7.08	41	0.54	0.68	0.21
	FA-6	28	12	0	27	92.31%	7.7	6.5	7.23	NA	33	0.5	0.6	0.19 *
	LT-3	4	4	0	3.77	76.92%	3.15	NA	NA	NA	4	0.53	0.50	0.10 *
*M. zoquepopolucae*	TOTAL	15	9	0	14.69	100%	6.46	NA	8.00	6.39	8	0.68	0.6	−0.01
	FA-3	5	2	0	5	92.31%	3.85	NA	NA	NA	1	0.69	0.58	−0.08
	FA-4	3	2	0	3	84.62%	3.23	NA	NA	NA	2	0.72	0.54	−0.14
	FA-14	4	3	0	4	92.31%	3.7	NA	NA	NA	0	0.65	0.58	0.01
	LT-5	3	2	0	2.692	84.62%	2.92	NA	NA	NA	1	0.67	0.54	0.01

**Table 5:** Details of the localities collected for each species for *Magnolia* in the states of Puebla and Veracruz, Mexico. Locality coordinates have been omitted because of conservation concerns but can be obtained from the corresponding author. Voucher specimens will be deposited in the herbaria IEB, MEXU, and XAL (acronyms are according to [86]). ^1^ Locality was a seedling nursery. N: Sample size.

Species	Locality	State	Municipality	N	Voucher
*M. decastroi*	FA-12	Veracruz	Zongolica	30	NA ^1^
	FA-13			33	Aldaba 224
*M. lopezobradorii*	FA-5	Veracruz	San Andrés Tuxtla	3	Aldaba 241
	FA-7			27	Aldaba 242
	FA-8			3	Aldaba 245
	LT-2			21	Samain & Martínez 2016–03
*M. mexicana*	FA-2	Puebla	Cuetzalan del Progreso	13	Aldaba 215
	FA-10		Xicotepec	31	Aldaba 219
	FA-11		Hueytamalco	4	Aldaba 202
	FA-1	Veracruz	Xalapa	24	Aldaba 210
	FA-9		Yecuatla	7	Aldaba 218
	FA-15		Coatepec	18	Aldaba 227
*M. sinacacolinii*	FA-6	Veracruz	Catemaco	29	Aldaba 235
	LT-3		San Andrés Tuxtla	4	Samain & Martínez 2016-07
*M. zoquepopolucae*	FA-3	Veracruz	Soteapan	5	Aldaba 239
	FA-4			3	Aldaba 240
	FA-14			4	Aldaba 247
	LT-5			3	Samain & Martínez 2016–12

## Data Availability

The data presented in this study are available on request from the corresponding author. The data is not publicly available do its usage in the ongoing study.
